# Retraction: CircRNA_0058063 functions as a ceRNA in bladder cancer progression via targeting miR-486-3p/FOXP4 axis

**DOI:** 10.1042/BSR20193484_RET

**Published:** 2026-05-06

**Authors:** Haote Liang, Hang Huang, Yeping Li, Yongyong Lu, Tingyu Ye

**Affiliations:** Department of Urology, The First Affiliated Hospital of Wenzhou Medical University, Wenzhou 325000, Zhejiang Province, China

**Keywords:** biomarker, Bladder cancer (BC), circRNA_0058063, miR-486-3p, progression

This article is being retracted from *Bioscience Reports* at the request of the Editor-in-Chief and the Editorial Board. This follows the receipt of a notification from a reader, alerting the Editorial Board to similarities between their flow cytometry plots in Figure 2D, compared to another author group's published article.

Specifically, these similarities include:

- The miR-NC panel from Figure 5F of DOI: 10.1016/j.canlet.2017.06.027 appears highly similar to that of Figure 2D 5637/si-NC panel of DOI 10.1042/bsr20193484
- The miR-29a panel from Figure 5F of DOI: 10.1016/j.canlet.2017.06.027 appears highly similar to that of Figure 2D 5637/si-circRNA_0058063 panel of DOI 10.1042/bsr20193484
- The miR-29a + pVax1 panel from Figure 5F of DOI: 10.1016/j.canlet.2017.06.027appears highly similar to that of Figure 2D BIU-87/si-circRNA_0058063 panel of DOI 10.1042/bsr20193484
- The miR-29a + pVax1-VEGFA pVax1 panel from Figure 5F of DOI: 10.1016/j.canlet.2017.06.027 appears highly similar to that of Figure 2D BIU-87/si-NC panel of DOI 10.1042/bsr20193484

Additionally, the background details of the ‘raw’ Western Blots which appear to be edited when compared to the Figures themselves

The authors have cooperated with the investigation, but are unable to provide the original data for the article or address any of the concerns raised. Given the extent of the issues raised, the Editorial Board stands by the decision to retract the article. The authors, Editor-in-Chief and Editorial Board agree with the retraction.

